# Supplementary figures and images for: Differences in the endophytic fungal community and effective ingredients in root of three Glycyrrhiza species in Xinjiang, China
Source: PeerJ. 2021 Mar 9;9:e11047. doi: 10.7717/peerj.11047 (PMC7953873; doi:10.7717/peerj.11047)

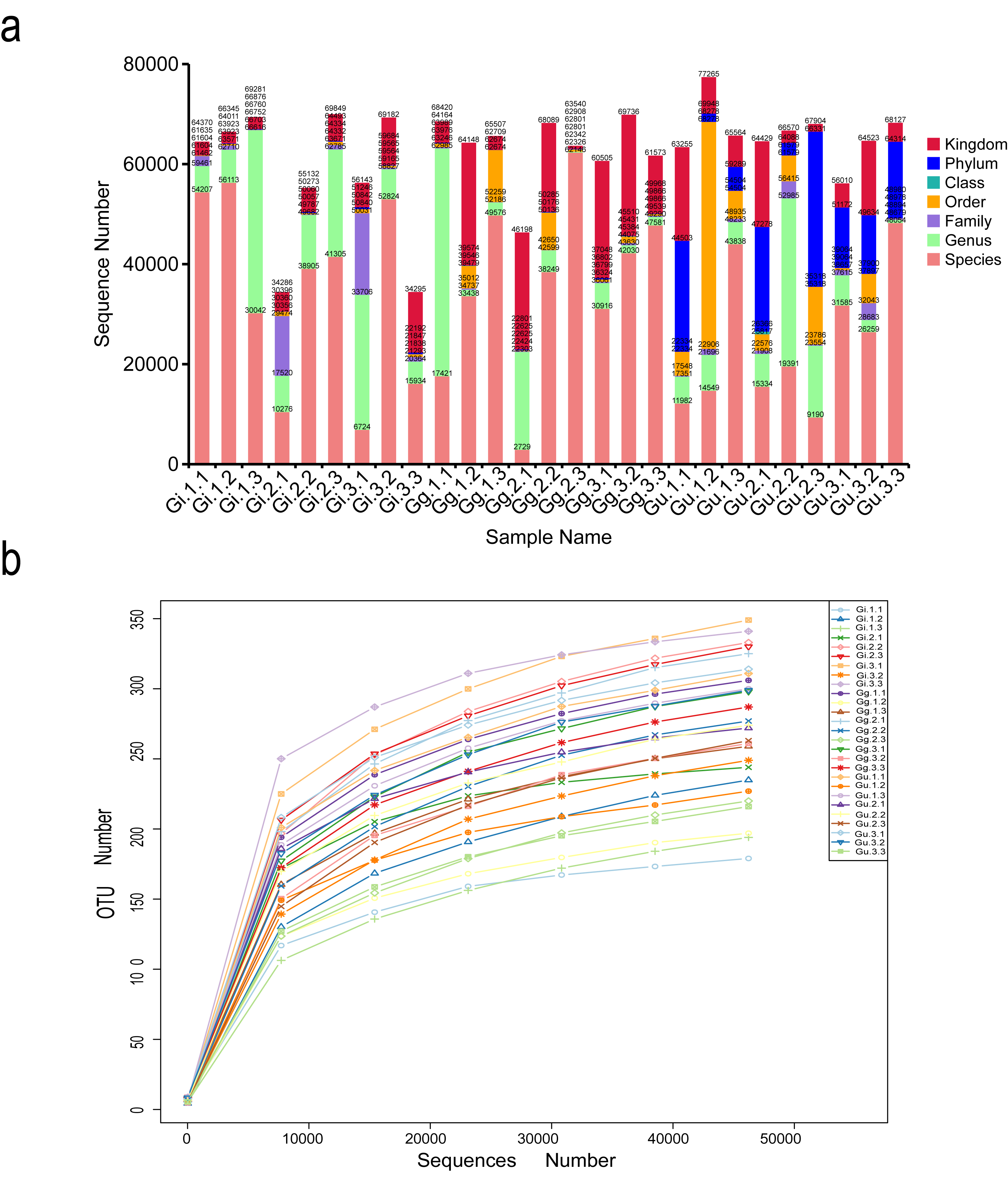

Supplement: Supplemental Information 1 — Sample Name: Gi, Gg and Gu: Glycyrrhiza inflata, Glycyrrhiza glabra and Glycyrrhiza uralensis; 1, 2 and 3: root depth 0-20 cm, 20-40 cm, and 40-60 cm, respectively; the third number representing the replicate number. The rarefaction curves different colors represent different samples. [file peerj-09-11047-s001.png]
