# Supplementary material for: Differences in the endophytic fungal community and effective ingredients in root of three Glycyrrhiza species in Xinjiang, China
Source: PeerJ. 2021 Mar 9;9:e11047. doi: 10.7717/peerj.11047 (PMC7953873; doi:10.7717/peerj.11047)
Supplement: Supplemental Information 2 [file peerj-09-11047-s002.docx]

**Table S1.** Geographical sources and physicochemical properties of soil samples

| **Sampling Site** | **Yiwu County, Xinjiang Province** | **Hami City, Xinjiang Province** | **Shihezi City, Xinjiang Province** |
| --- | --- | --- | --- |
| Plant Species | *Glycyrrhiza uralensis* | *Glycyrrhiza inflata* | *Glycyrrhiza glabra* |
| Altitude (m) | 1372.8 | 806.1 | 340.2 |
| Latitude and longitude | 43°33′58″N, 94°81′86″E | 42°84′48″N, 93°54′80″E | 44°45′18″N, 86°06′39″E |
| Annual average temperature (°C) | 5.5 | 9.8 | 8.1 |
| Annual average precipitation (mm) | 105.8 | 33.8 | 225 |
| Total nitrogen (g/kg) | 0.832 | 0.762 | 0.693 |
| Total phosphorus (g/kg) | 0.712 | 0.537 | 0.665 |
| Total potassium(g/kg) | 19.743 | 21.864 | 20.771 |
| PH | 8.534 | 8.45 | 8.831 |
| Soil water content (%) | 3.58 | 4.92 | 7.98 |
| organic matter (g/kg) | 14.744 | 27.99 | 10.495 |
| total salt (g/kg) | 1.033 | 5.697 | 4.894 |
| nitrate nitrogen (mg/kg) | 7.592 | 14.2 | 3.552 |
| ammonium nitrogen (mg/kg) | 6.021 | 4.869 | 3.326 |
| Available phosphorus (mg/kg) | 3.699 | 9.677 | 5.292 |
| Available potassium (mg/kg) | 81.208 | 180.032 | 273.093 |
