# Supplementary material for: Differences in the endophytic fungal community and effective ingredients in root of three Glycyrrhiza species in Xinjiang, China
Source: PeerJ. 2021 Mar 9;9:e11047. doi: 10.7717/peerj.11047 (PMC7953873; doi:10.7717/peerj.11047)
Supplement: Supplemental Information 3 — Raw data of the effective ingredients and physicochemical properties of samples, Gi, Gg and Gu:Glycyrrhiza inflata,Glycyrrhiza glabra and Glycyrrhiza uralensis; 1, 2 and 3: root depth 0–20 cm, 20–40 cm, and 40–60 cm. Abbreviations: GlA, glycyrrhizic acid; GTF, total flavonoid; LI, liquiritin; SOM, soil organic matter; STN, soil total nitrogen; STP, soil total phosphorus; STK, soil total potassium; SNN, soil nitrate nitrogen; SAN, soil ammonium nitrogen; SAP, soil available phosphorus; SAK, soil available potassium; TS, total salt; PH, soil pH; SWC, soil water content. [file peerj-09-11047-s003.docx]

**Table S2.** The raw data of effective ingredients and physicochemical properties of samples

|  | **Variable** | **Gi1** | **Gi2** | **Gi3** | **Gg1** | **Gg2** | **Gg3** | **Gu1** | **Gu2** | **Gu3** |
| --- | --- | --- | --- | --- | --- | --- | --- | --- | --- | --- |
| Root effective ingredients | LI (%) | 0.618 | 1.188 | 1.089 | 0.643 | 1.086 | 1.093 | 1.446 | 1.849 | 2.068 |
|  | GlA (%) | 1.999 | 1.996 | 2.098 | 2.641 | 2.920 | 2.692 | 1.792 | 2.346 | 2.081 |
|  | GTF (%) | 4.554 | 4.604 | 4.408 | 4.549 | 4.639 | 4.559 | 4.568 | 4.575 | 4.647 |
| Soil physicochemical properties | SOM (g/kg) | 27.846 | 28.384 | 27.740 | 11.711 | 11.873 | 7.903 | 15.888 | 13.997 | 14.349 |
|  | STN (g/kg) | 0.939 | 0.748 | 0.600 | 0.881 | 0.694 | 0.504 | 0.707 | 0.907 | 0.882 |
|  | STP (g/kg) | 0.563 | 0.545 | 0.503 | 0.679 | 0.680 | 0.638 | 0.717 | 0.708 | 0.711 |
|  | STK (g/kg) | 21.553 | 21.304 | 22.735 | 20.878 | 20.647 | 20.787 | 19.695 | 19.539 | 19.995 |
|  | SNN (mg/kg) | 20.558 | 14.059 | 7.983 | 4.666 | 3.217 | 2.773 | 7.899 | 8.027 | 6.851 |
|  | SAN (mg/kg) | 5.315 | 5.301 | 3.991 | 3.263 | 3.152 | 3.562 | 6.168 | 5.631 | 6.263 |
|  | SAP (mg/kg) | 11.562 | 10.732 | 6.736 | 7.623 | 5.378 | 2.874 | 3.741 | 4.136 | 3.221 |
|  | SAK (mg/kg) | 207.016 | 161.299 | 171.779 | 276.738 | 256.856 | 285.684 | 88.775 | 79.063 | 75.784 |
|  | TS (g/kg) | 8.492 | 6.117 | 2.483 | 4.008 | 4.267 | 6.408 | 1.125 | 0.975 | 1.000 |
|  | PH | 8.477 | 8.343 | 8.530 | 8.807 | 8.893 | 8.793 | 8.480 | 8.570 | 8.553 |
|  | SWC (%) | 4.480 | 4.937 | 5.353 | 6.950 | 7.647 | 9.353 | 2.910 | 3.537 | 4.303 |

Description: Raw data of the effective ingredients and physicochemical properties of samples, Gi, Gg and Gu: *Glycyrrhiza inflata*, *Glycyrrhiza glabra* and *Glycyrrhiza uralensis*; 1, 2 and 3: root depth 0-20cm, 20-40cm, and 40-60cm. Abbreviations: GlA, glycyrrhizic acid; GTF, total flavonoid; LI, liquiritin; SOM, soil organic matter; STN, soil total nitrogen; STP, soil total phosphorus; STK, soil total potassium; SNN, soil nitrate nitrogen; SAN, soil ammonium nitrogen; SAP, soil available phosphorus; SAK, soil available potassium; TS, total salt; PH, soil pH; SWC, soil water content.
