# Supplementary material for: Differences in the endophytic fungal community and effective ingredients in root of three Glycyrrhiza species in Xinjiang, China
Source: PeerJ. 2021 Mar 9;9:e11047. doi: 10.7717/peerj.11047 (PMC7953873; doi:10.7717/peerj.11047)
Supplement: Supplemental Information 4 — P < 0.05 indicates statistical significance. [file peerj-09-11047-s004.docx]

**Table S3** Effect of plant species and root depth on the bioactive compounds of licorice root

| **Source** | **Dependent variable** | **Type III Sum of Squares** | **Degrees of freedom** | **Mean Square** | **F** | ***p* value** | **Partial Eta Squared** |
| --- | --- | --- | --- | --- | --- | --- | --- |
| Plant species | GIA | 2.939 | 2 | 1.469 | 3.554 | 0.050 | 0.283 |
|  | GTF | 0.028 | 2 | 0.014 | 0.201 | 0.820 | 0.022 |
|  | LI | 4.183 | 2 | 2.091 | 4.763 | **0.022** | 0.346 |
| root depth | GIA | 0.344 | 2 | 0.172 | 0.416 | 0.666 | 0.044 |
|  | GTF | 0.022 | 2 | 0.011 | 0.158 | 0.855 | 0.017 |
|  | LI | 1.466 | 2 | 0.733 | 1.670 | 0.216 | 0.156 |
| Species*root depth | GIA | 0.268 | 4 | 0.067 | 0.162 | 0.955 | 0.035 |
|  | GTF | 0.066 | 4 | 0.016 | 0.233 | 0.916 | 0.049 |
|  | LI | 0.085 | 4 | 0.021 | 0.049 | 0.995 | 0.011 |

Description: *P*<0.05 indicates statistical significance.
