# Supplementary material for: Differences in the endophytic fungal community and effective ingredients in root of three Glycyrrhiza species in Xinjiang, China
Source: PeerJ. 2021 Mar 9;9:e11047. doi: 10.7717/peerj.11047 (PMC7953873; doi:10.7717/peerj.11047)
Supplement: Supplemental Information 5 — Raw reads refers to the sequence filtering out low-quality bases; clean reads refers to the sequence finally used for subsequent analysis after filtering chimeras; base refers to the number of bases of final clean reads; Avglen refers to the average length of clean reads. Q20 refers to the percentage of bases whose quality value is greater than 20 (sequencing error rate is less than 1%); GC (%) refers to the content of GC bases in clean reads; effective (%) refers to the percentage of the number of clean reads and the number of raw reads. Sample name: Gu, Gg and Gi:Glycyrrhiza uralensis,Glycyrrhiza glabra and Glycyrrhiza inflata, respectively; the second number representing root depth 1, 2 and 3: 0–20 cm, 20–40 cm, and 40–60 cm, respectively; the third number representing the replicate number. [file peerj-09-11047-s005.docx]

**Supplementary table S4** Sequencing results of each sample

| **Sample name** | **Raw reads** | **Clean Reads** | **Base (nt)** | **AvgLen (nt)** | **Q20** | **GC %** | **Effective %** |
| --- | --- | --- | --- | --- | --- | --- | --- |
| Gi.1.1 | 82142 | 80098 | 18625453 | 232 | 69.240 | 48.470 | 97.510 |
| Gi.1.2 | 82302 | 80156 | 19220714 | 239 | 72.440 | 42.210 | 97.390 |
| Gi.1.3 | 80434 | 80073 | 19542040 | 244 | 74.880 | 49.500 | 99.550 |
| Gi.2.1 | 55552 | 54262 | 14094949 | 259 | 81.270 | 52.800 | 97.680 |
| Gi.2.2 | 74017 | 71457 | 17186486 | 240 | 72.780 | 49.370 | 96.540 |
| Gi.2.3 | 82970 | 80116 | 20391135 | 254 | 81.930 | 45.790 | 96.560 |
| Gi.3.1 | 82448 | 80055 | 21979682 | 274 | 74.960 | 51.120 | 97.100 |
| Gi.3.2 | 81030 | 80117 | 19556235 | 244 | 80.420 | 43.040 | 98.870 |
| Gi.3.3 | 81622 | 80142 | 22802652 | 284 | 72.450 | 52.060 | 98.190 |
| Gg.1.1 | 81158 | 80078 | 20010631 | 249 | 80.870 | 48.340 | 98.670 |
| Gg.1.2 | 72594 | 71727 | 17065030 | 237 | 84.190 | 53.430 | 98.810 |
| Gg.1.3 | 82722 | 80158 | 18948038 | 236 | 74.460 | 50.000 | 96.900 |
| Gg.2.1 | 70596 | 66817 | 14835033 | 222 | 71.490 | 39.220 | 94.650 |
| Gg.2.2 | 82567 | 80068 | 19324183 | 241 | 80.120 | 53.470 | 96.970 |
| Gg.2.3 | 84732 | 80088 | 18548845 | 231 | 71.670 | 48.750 | 94.520 |
| Gg.3.1 | 82448 | 80161 | 22910413 | 285 | 73.380 | 45.910 | 97.230 |
| Gg.3.2 | 82613 | 80134 | 19304222 | 240 | 84.140 | 53.530 | 97.000 |
| Gg.3.3 | 84910 | 80144 | 18620869 | 232 | 73.800 | 49.380 | 94.390 |
| Gu.1.1 | 88740 | 80119 | 18884241 | 235 | 78.140 | 48.600 | 90.290 |
| Gu.1.2 | 92042 | 88936 | 21094384 | 237 | 80.940 | 47.210 | 96.630 |
| Gu.1.3 | 86285 | 80095 | 19248782 | 240 | 76.770 | 48.840 | 92.830 |
| Gu.2.1 | 89040 | 83098 | 20596345 | 247 | 74.400 | 46.480 | 93.330 |
| Gu.2.2 | 85607 | 80073 | 19898465 | 248 | 80.040 | 48.680 | 93.540 |
| Gu.2.3 | 83398 | 80106 | 20772062 | 259 | 81.220 | 48.420 | 96.050 |
| Gu.3.1 | 82648 | 80086 | 20755064 | 259 | 72.160 | 44.810 | 96.900 |
| Gu.3.2 | 83175 | 80051 | 20078993 | 250 | 79.420 | 48.810 | 96.240 |
| Gu.3.3 | 81356 | 80218 | 19558935 | 243 | 82.630 | 49.320 | 98.600 |

Description: Raw reads refers to the sequence filtering out low-quality bases; clean reads refers to the sequence finally used for subsequent analysis after filtering chimeras; base refers to the number of bases of final clean reads; Avglen refers to the average length of clean reads. Q20 refers to the percentage of bases whose quality value is greater than 20 (sequencing error rate is less than 1%); GC (%) refers to the content of GC bases in clean reads; effective (%) refers to the percentage of the number of clean reads and the number of raw reads. Sample name: Gu, Gg and Gi: *Glycyrrhiza uralensis*, *Glycyrrhiza glabra* and *Glycyrrhiza inflata*, respectively; the second number representing root depth 1, 2 and 3: 0-20cm, 20-40cm, and 40-60cm, respectively; the third number representing the replicate number.
