# Supplementary material for: Differences in the endophytic fungal community and effective ingredients in root of three Glycyrrhiza species in Xinjiang, China
Source: PeerJ. 2021 Mar 9;9:e11047. doi: 10.7717/peerj.11047 (PMC7953873; doi:10.7717/peerj.11047)
Supplement: Supplemental Information 6 — Community richness was identified using the Chao1 and ACE estimator. Community diversity was identified using the Shannon and Simpson indexes. Sequencing depth was characterized by Good’s coverage, good’s coverage estimator values was 99.9%, indicating that the sequence numbers per sample were high enough and has met the requirements. Sample name: Gi, Gg and Gu:Glycyrrhiza inflata,Glycyrrhiza glabra and Glycyrrhiza uralensis; 1, 2 and 3: root depth 0–20 cm, 20–40 cm, and 40–60 cm. [file peerj-09-11047-s006.docx]

**Table S5** The alpha diversity indexes in each group

| **Sample name** | **Observed species** | **Community diversity** | | **Community richness** | | **Goods coverage** | **PD-whole tree** |
| --- | --- | --- | --- | --- | --- | --- | --- |
|  |  | **Shannon** | **Simpson** | **Chao1** | **ACE** |  |  |
| Gi1 | 202.667 | 3.393 | 0.747 | 238.678 | 253.105 | 0.999 | 35.233 |
| Gi2 | 302.333 | 4.678 | 0.907 | 327.516 | 338.868 | 0.999 | 63.772 |
| Gi3 | 313.000 | 4.218 | 0.843 | 356.317 | 355.694 | 0.999 | 73.547 |
| Gg1 | 254.000 | 3.897 | 0.829 | 282.714 | 292.674 | 0.999 | 51.440 |
| Gg2 | 274.000 | 3.736 | 0.821 | 304.665 | 316.987 | 0.999 | 50.496 |
| Gg3 | 282.000 | 4.060 | 0.826 | 320.275 | 329.183 | 0.999 | 48.561 |
| Gu1 | 279.333 | 4.910 | 0.911 | 317.592 | 323.995 | 0.999 | 38.791 |
| Gu2 | 269.667 | 4.246 | 0.863 | 303.285 | 312.849 | 0.999 | 40.653 |
| Gu3 | 276.333 | 4.470 | 0.883 | 326.368 | 324.554 | 0.999 | 49.658 |

Description: Community richness was identified using the Chao1 and ACE estimator. Community diversity was identified using the Shannon and Simpson indexes. Sequencing depth was characterized by Good’s coverage, good’s coverage estimator values was 99.9%, indicating that the sequence numbers per sample were high enough and has met the requirements. Sample name: Gi, Gg and Gu: *Glycyrrhiza inflata*, *Glycyrrhiza glabra* and *Glycyrrhiza uralensis*; 1, 2 and 3: root depth 0-20cm, 20-40cm, and 40-60cm.
