# Supplementary material for: Differences in the endophytic fungal community and effective ingredients in root of three Glycyrrhiza species in Xinjiang, China
Source: PeerJ. 2021 Mar 9;9:e11047. doi: 10.7717/peerj.11047 (PMC7953873; doi:10.7717/peerj.11047)
Supplement: Supplemental Information 7 — Others: The sum of the undefined and unannotated parts. [file peerj-09-11047-s007.docx]

**Table S6.** Composition of dominant fungi at each classification level

|  | **Taxonomy** | **Gi1** | **Gi2** | **Gi3** | **Gg1** | **Gg2** | **Gg3** | **Gu1** | **Gu2** | **Gu3** |
| --- | --- | --- | --- | --- | --- | --- | --- | --- | --- | --- |
| **Class** | Sordariomycetes | 28.543% | 19.304% | 3.157% | 43.197% | 51.607% | 50.469% | 32.480% | 8.405% | 23.550% |
|  | Dothideomycetes | 57.528% | 7.810% | 27.693% | 33.895% | 9.526% | 1.062% | 13.503% | 27.560% | 30.872% |
|  | Agaricomycetes | 3.775% | 21.177% | 27.926% | 1.915% | 0.676% | 12.487% | 0.582% | 6.433% | 5.045% |
|  | Leotiomycetes | 0.099% | 13.314% | 0.583% | 0.968% | 0.229% | 0.922% | 19.077% | 8.191% | 1.589% |
|  | Tremellomycetes | 0.042% | 0.068% | 0.469% | 0.984% | 9.945% | 0.031% | 0.022% | 0.045% | 0.059% |
|  | Pezizomycetes | 3.795% | 15.260% | 5.674% | 0.056% | 0.238% | 0.120% | 0.172% | 0.536% | 0.172% |
|  | Eurotiomycetes | 1.338% | 3.115% | 0.387% | 0.757% | 0.230% | 0.805% | 2.292% | 10.110% | 3.523% |
|  | Saccharomycetes | 0.087% | 1.505% | 1.656% | 0.683% | 0.238% | 0.643% | 0.076% | 0.243% | 0.295% |
|  | Glomeromycetes | 0.002% | 0.588% | 0.322% | 0.172% | 0.036% | 0.346% | 0.043% | 0.043% | 0.151% |
|  | Olpidiomycetes | 0.000% | 0.002% | 0.002% | 0.394% | 0.007% | 0.001% | 0.004% | 0.010% | 0.001% |
|  | Others | 4.792% | 17.858% | 32.132% | 16.980% | 27.269% | 33.116% | 31.751% | 38.426% | 34.744% |
| **Older** | Hypocreales | 28.147% | 14.167% | 2.529% | 42.964% | 50.316% | 50.297% | 29.152% | 7.565% | 23.456% |
|  | Pleosporales | 57.207% | 1.351% | 24.898% | 31.457% | 8.509% | 0.893% | 13.336% | 27.125% | 15.693% |
|  | Thelephorales | 3.544% | 9.351% | 23.338% | 0.048% | 0.112% | 0.062% | 0.068% | 1.529% | 1.370% |
|  | Helotiales | 0.095% | 13.305% | 0.446% | 0.099% | 0.118% | 0.074% | 19.077% | 8.188% | 1.584% |
|  | Capnodiales | 0.168% | 6.446% | 2.721% | 2.403% | 0.997% | 0.126% | 0.157% | 0.433% | 15.177% |
|  | Agaricales | 0.038% | 11.061% | 3.924% | 1.536% | 0.220% | 12.202% | 0.420% | 1.131% | 2.379% |
|  | Filobasidiales | 0.016% | 0.058% | 0.262% | 0.109% | 9.940% | 0.016% | 0.020% | 0.017% | 0.053% |
|  | Pezizales | 3.794% | 15.258% | 5.674% | 0.056% | 0.238% | 0.120% | 0.172% | 0.255% | 0.172% |
|  | Chaetothyriales | 1.234% | 2.796% | 0.063% | 0.557% | 0.134% | 0.671% | 1.890% | 9.733% | 3.409% |
|  | Glomerellales | 0.371% | 4.905% | 0.122% | 0.050% | 0.008% | 0.000% | 0.012% | 0.008% | 0.069% |
|  | Others | 5.387% | 21.302% | 36.022% | 20.721% | 29.409% | 35.540% | 35.696% | 44.016% | 36.640% |
| **Family** | Nectriaceae | 27.921% | 8.655% | 1.247% | 24.036% | 32.103% | 25.407% | 21.887% | 6.199% | 19.770% |
|  | Phaeosphaeriaceae | 27.742% | 0.314% | 23.942% | 0.104% | 0.172% | 0.140% | 2.564% | 4.848% | 14.200% |
|  | Massarinaceae | 26.567% | 0.321% | 0.196% | 25.124% | 3.964% | 0.194% | 8.224% | 17.408% | 0.736% |
|  | Thelephoraceae | 3.544% | 9.351% | 23.338% | 0.048% | 0.112% | 0.062% | 0.068% | 1.529% | 1.370% |
|  | Hypocreales-fam-Incertae-sedis | 0.182% | 3.459% | 0.159% | 16.890% | 17.667% | 24.240% | 4.379% | 0.817% | 1.504% |
|  | Cladosporiaceae | 0.164% | 6.446% | 2.721% | 2.403% | 0.997% | 0.125% | 0.157% | 0.426% | 15.174% |
|  | Helotiales-fam-Incertae-sedis | 0.038% | 13.200% | 0.092% | 0.016% | 0.051% | 0.023% | 0.257% | 0.099% | 0.038% |
|  | Bolbitiaceae | 0.008% | 0.065% | 0.049% | 1.489% | 0.108% | 12.068% | 0.016% | 0.034% | 0.038% |
|  | Psathyrellaceae | 0.014% | 10.917% | 3.833% | 0.025% | 0.108% | 0.021% | 0.044% | 1.014% | 2.341% |
|  | Filobasidiaceae | 0.016% | 0.058% | 0.094% | 0.103% | 9.937% | 0.011% | 0.005% | 0.013% | 0.014% |
|  | Others | 13.805% | 47.214% | 44.329% | 29.764% | 34.781% | 37.710% | 62.399% | 67.614% | 44.816% |
| **Species** | *Fusarium-solani* | 27.464% | 8.159% | 0.575% | 23.412% | 31.622% | 21.859% | 17.792% | 3.779% | 17.460% |
|  | *Paraphoma-radicina* | 27.738% | 0.308% | 23.937% | 0.102% | 0.171% | 0.136% | 1.964% | 4.485% | 13.980% |
|  | *Sarocladium-kiliense* | 0.164% | 3.105% | 0.111% | 16.547% | 17.243% | 21.897% | 4.218% | 0.697% | 1.302% |
|  | *Cladosporium-chasmanthicola* | 0.158% | 1.469% | 2.681% | 0.099% | 0.827% | 0.097% | 0.142% | 0.376% | 15.147% |
|  | *Cadophora-malorum* | 0.038% | 13.200% | 0.092% | 0.016% | 0.051% | 0.023% | 0.257% | 0.099% | 0.038% |
|  | *Psathyrella-candolleana* | 0.014% | 10.917% | 3.833% | 0.025% | 0.108% | 0.021% | 0.004% | 1.014% | 2.341% |
|  | *Conocybe-velutipes* | 0.007% | 0.055% | 0.025% | 1.018% | 0.100% | 10.447% | 0.014% | 0.034% | 0.034% |
|  | *Helminthosporium-solani* | 7.493% | 0.061% | 0.032% | 1.747% | 0.158% | 0.030% | 2.228% | 2.887% | 0.195% |
|  | *Cladosporium-cladosporioides* | 0.005% | 4.885% | 0.009% | 2.189% | 0.032% | 0.021% | 0.006% | 0.045% | 0.024% |
|  | *Fusarium-sacchari* | 0.276% | 0.397% | 0.454% | 0.449% | 0.431% | 3.463% | 0.320% | 0.269% | 0.686% |
|  | Others | 36.641% | 57.446% | 68.252% | 54.398% | 49.259% | 42.006% | 73.055% | 86.316% | 48.793% |

Description: Others: The sum of the undefined and unannotated parts.
