# Supplementary material for: Differences in the endophytic fungal community and effective ingredients in root of three Glycyrrhiza species in Xinjiang, China
Source: PeerJ. 2021 Mar 9;9:e11047. doi: 10.7717/peerj.11047 (PMC7953873; doi:10.7717/peerj.11047)
Supplement: Supplemental Information 8 — r2 is the determinant coefficients of the distribution of the fungal community by environmental factors. Abbreviations: GlA, glycyrrhizic acid; GTF, total flavonoid; LI, liquiritin; SOM, soil organic matter; STN, soil total nitrogen; STP, soil total phosphorus; STK, soil total potassium; SNN, soil nitrate nitrogen; SAN, soil ammonium nitrogen; SAP, soil available phosphorus; SAK, soil available potassium; TS, total salt; PH, soil pH; SWC, soil water content. [file peerj-09-11047-s008.docx]

**Table S7.** Results for db-RDA testing effects of soil physicochemical properties and bioactive compounds on the composition and distribution of fungal community in licorice root.

|  | ***r*^2^** | ***Pr* (>r)** |
| --- | --- | --- |
| SOM | 0.202 | 0.071 |
| STN | 0.089 | 0.329 |
| STP | 0.099 | 0.291 |
| STK | 0.135 | 0.174 |
| SNN | 0.175 | 0.100 |
| SAN | 0.231 | 0.044 |
| SAP | 0.070 | 0.423 |
| SAK | 0.329 | 0.008 |
| TS | 0.099 | 0.271 |
| PH | 0.053 | 0.524 |
| SWC | 0.121 | 0.220 |
| RWC | 0.247 | 0.027 |
| GlA | 0.026 | 0.730 |
| GTF | 0.038 | 0.634 |
| LI | 0.243 | 0.034 |

Description: *r*^2^ is the determinant coefficients of the distribution of the fungal community by environmental factors. Abbreviations: GlA, glycyrrhizic acid; GTF, total flavonoid; LI, liquiritin; SOM, soil organic matter; STN, soil total nitrogen; STP, soil total phosphorus; STK, soil total potassium; SNN, soil nitrate nitrogen; SAN, soil ammonium nitrogen; SAP, soil available phosphorus; SAK, soil available potassium; TS, total salt; PH, soil pH; SWC, soil water content.
